# A modified *p53* enhances apoptosis in sarcoma cell lines mediated by doxorubicin

**DOI:** 10.1038/sj.bjc.6601653

**Published:** 2004-02-24

**Authors:** H-J Tang, D Qian, V K Sondak, S Stachura, J Lin

**Affiliations:** 1 Department of Obstetrics and Gynecology, University of Michigan Comprehensive Cancer Center, Ann Arbor, MI 48109, USA; 2 Department of Internal Medicine, University of Michigan Comprehensive Cancer Center, Ann Arbor, MI 48109, USA; 3 Department of Surgery, University of Michigan Comprehensive Cancer Center, Ann Arbor, MI 48109, USA

**Keywords:** p53, Mdm2, doxorubicin, cisplatin, apoptosis, sarcoma cell lines

## Abstract

Mdm2 is frequently overexpressed in sarcoma cells and may contribute to drug resistance by increasing p53 degradation. We investigated the induction of apoptosis in sarcoma cells via adenovirus-mediated gene transfer of wild-type *p53* and two modified *p53* genes, *p53* 14/19 and *p53* 22/23, whose protein products are resistant to Mdm2-mediated degradation. We found that adenovirus-wt *p53* (Ad-wt *p53*) induces significant apoptosis in HT1080 fibrosarcoma cells expressing low levels of Mdm2, but fails to induce apoptosis in SJSA osteosarcoma cells expressing high levels of Mdm2. In contrast, Ad-*p53* 14/19 induces significant apoptosis in both cell lines. Interestingly, Ad-*p53* 22/23, a vector encoding a transcription-defective p53 mutant, causes limited apoptosis in both cell lines. We demonstrate that doxorubicin induces phosphorylation of both wt p53 and p53 14/19 protein at multiple sites. We tested the efficacy of doxorubicin and cisplatin with either Ad-wt *p53*, Ad-*p53* 22/23 or Ad-*p53* 14/19. SJSA cells, although harbouring endogenous wt *p53*, did not undergo significant apoptosis following doxorubicin or cisplatin exposure alone or combined with Ad-wt *p53*. In contrast, doxorubicin or cisplatin plus Ad-*p53* 14/19 induced significant apoptosis. Gene transfer of *p53* 14/19 in combination with the administration of doxorubicin or cisplatin is a potential therapeutic approach for cancers expressing high levels of Mdm2.

Sarcomas are uncommon malignancies generally arising from connective tissue or bone. Although these neoplasms are rare, important insights have been gained from studying their biology, and have led to new therapeutic approaches (Joensuu *et al*, 2001). One such discovery is the fundamental role of the p53/Mdm2 pathway.

The *p53* tumour suppressor gene encodes a transcription factor that plays a critical role in regulating the cell cycle and maintaining genomic integrity by inducing growth arrest, DNA repair and apoptosis in response to a variety of stresses (Vogelstein and Kinzler, 1992; Donehower and Bradley, 1993; Prives and Hall, 1999). Functional inactivation of p53 is a common event in the development of human cancer (Levine *et al*, 1991; Vogelstein *et al*, 2000), and p53 inactivation is typically caused by p53 mutations, Mdm2 overexpression and other mechanisms. It is well known that more than 50% of all human cancers contain p53 mutations. To target the p53 mutations in human cancers, ONYX-015 (*dl*1520), which selectively replicates in and kills p53-deficient cells, is being explored as an anti-tumour agent in clinical trials (Hann and Balmain, 2003).

Mdm2, a major negative regulator of p53, inactivates p53 protein by binding its transcriptional activation domain, inhibiting p53's regulation of target genes and its antiproliferative effects (Chen *et al*, 1996; Haupt *et al*, 1997a; Kubbutat *et al*, 1997, 1998). Mdm2 is the major p53 E3 ubiquitin ligase that governs ubiquitination and degradation of p53 (Haupt *et al*, 1997a; Honda *et al*, 1997; Kubbutat *et al*, 1997). *mdm2* amplification is common in sarcomas, and most of these tumours retain wt *p53* (Leach *et al*, 1993; Keleti *et al*, 1996), suggesting that tumours with Mdm2 overexpression bypass the need to mutate *p53*.

Loss of p53 function contributes not only to tumour progression but also to resistance of tumours to chemotherapy or radiation therapy (Lowe *et al*, 1993). Overexpression of Mdm2 can confer resistance to cytotoxic drugs (Kondo *et al*, 1995, 1996; Suzuki *et al*, 1998). Mdm2 has also been shown to induce expression of the multidrug resistance 1 (*mdr1*) gene and its main product P-glycoprotein (P-gp) (Kondo *et al*, 1996), which has been implicated in chemoresistance in human sarcomas (Chan *et al*, 1990; Hoffman *et al*, 1999; Jiminez *et al*, 1999). Wild-type *p53* can sensitise sarcoma cells harbouring *p53* mutations to doxorubicin by downregulating MDR-1 and P-gp expression (Zhan *et al*, 2001).

Restoration of normal p53 function has been evaluated as a strategy for cancer therapy (Beaudry *et al*, 1996). The most direct approach involves transfer of wt *p53* gene to cancer cells that lack endogenous p53 function (Yang *et al*, 1995; Zhang *et al*, 1995; Ko *et al*, 1996). However, since many sarcomas overexpress Mdm2, and high levels of Mdm2 inactivate and cause degradation of p53 (Haupt *et al*, 1997a; Momand *et al*, 1992; Oliner *et al*, 1992, 1993; Nakayama *et al*, 1995; Momand *et al*, 1998; Vogelstein *et al*, 2000), wt *p53* transfer into sarcoma cells that overexpress Mdm2 is unlikely to be efficacious (Chen *et al*, 1996; Meng *et al*, 1998).

To overcome the inhibition of p53 by Mdm2, two modified *p53* genes were constructed, *p53* 14/19 and *p53* 22/23, which contain double substitutions at residues Leu-14; Phe-19, and Leu-22; Trp-23 (Lin *et al*, 1994, 2000). As p53 14/19 and p53 22/23 proteins are defective in the ability to bind Mdm2, they are completely resistant to Mdm2-mediated degradation (Chen *et al*, 1995, 1996; Haupt *et al*, 1997a). In this study, we evaluate induction of apoptosis by adenovirus-*p53* 14/19 (Ad-*p53* 14/19) and adenovirus-*p53* 22/23 (Ad-*p53* 22/23) in low and high Mdm2 sarcoma cell lines. The previous study indicated that high levels of Mdm2 might confer multidrug resistance (Cocker *et al*, 2001). Therefore, we also investigated the synergistic effect on apoptosis between Ad-wt *p53* or Ad-*p53* 14/19 and doxorubicin or cisplatin. We further examined the difference in transactivation function between Ad-*p53* 14/19 and Ad-wt *p53* when combined with doxorubicin in an Mdm2 overexpressing sarcoma cell line.

## MATERIALS AND METHODS

### Cell lines and cell culture

SJSA osteosarcoma cells (provided by Jiandong Chen, H Lee Moffitt Cancer Center), HT1080 fibrosarcoma cells (American Type Culture Collection, Manassas, VA, USA) and normal human skin fibroblasts (NHF) that have a limited life span (provided by Mats Ljungman, University of Michigan Cancer Center) were used. SJSA cells exhibit amplification of *mdm2* and express high levels of Mdm2 protein (Oliner *et al*, 1992; Landers *et al*, 1997), whereas HT1080 cells express low levels of Mdm2 (Lin *et al*, 2000). Both cell lines express low levels of wt *p53* (Evdokiou and Cowled, 1998). Normal human skin fibroblasts were used as a control to assess the safety of adenovirus-mediated transfer of *p53* 14/19 *in vitro*. Cells were maintained in Dulbecco's modified Eagle's medium (DMEM, Gibco/BRL, Grand Island, NY, USA) containing 10% foetal bovine serum (FBS) and antibiotics (penicillin G 5000 U ml^−1^, streptomycin 5000 *μ*g ml^−1^, Gibco/BRL). Cells were grown as an attached monolayer at 37°C in a humidified atmosphere with 5% CO_2_.

### Adenovirus *p53* vector, doxorubicin and cisplatin

To generate the recombinant adenovirus-*p53* vectors, as described previously (Lin *et al*, 2000), cDNA for wt *p53*, *p53* 14/19 or *p53* 22/23 was cloned into an adenovirus vector, pACCMVpLpA(-)loxD (University of Michigan Vector Core). The negative control adenovirus (referred to as NCV, pACCMVpLpA(-)loxD) contains the same backbone as the other constructs. The human cytomegalovirus promoter was used to drive *p53* transcription for high level, constitutive expression. Doxorubicin (Calbiochem®, CN Biosciences, San Diego, CA, USA) was dissolved in water to produce a stock solution of 0.5 mg ml^−1^ and stored at 4°C. Cisplatin (American Pharmaceutical Partners, INS. Los Angeles) was diluted 1 mg per ml with 9 mg sodium chloride per ml in sterile water, and stored at room temperature.

### Apoptosis assay

To quantify apoptosis, 5 × 10^5^ cells/10-cm dish were infected by mock (no) infection, NCV, Ad-wt *p53*, Ad-*p53* 14/19 or Ad-*p53* 22/23 at a multiplicity of infection of 100–200 plaque forming units/cell in DMEM containing 2% FBS. The next day, the medium was removed and cells were rinsed twice with phosphate-buffered saline (PBS) to remove the adenovirus. Cells were then incubated in DMEM containing 10% FBS for 48 h. To assess synergy between Ad-wt *p53* or Ad-*p53* 14/19 and doxorubicin or cisplatin, some cells were also treated with 0.1 *μ*g ml^−1^ doxorubicin or 5 *μ*g ml^−1^ cisplatin, respectively. At 72 h after infection, both adherent and floating cells were harvested and fixed in 70% (vol/vol) ethanol. The cells were then stained with propidium iodide for 20 min in the dark. At least 1 × 10^5^ stained cells were analysed for Sub-G1 profile on a FACScan Flow Cytometer (Becton Dickinson, San Jose, CA, USA). The percentage of sub-G1 (apoptotic) cells in mock-infected cells and infected cells was calculated. The results presented are averages and standard deviations from three separate experiments.

### Western blot analysis

In order to compare transactivation function among Ad-*p53* 14/19, Ad-*p53* 22/23 and Ad-wt *p53*, levels of p53, Mdm2, p21^WAF-1^ and Bax were examined in SJSA and HT1080 cells (1.5 × 10^6^ cells/10-cm dish) infected by NCV, Ad-wt *p53*, Ad-*p53* 14/19 and Ad-*p53* 22/23. To analyse the levels of phosphorylated p53, phosphorylated Mdm2 and Bax, SJSA and HT1080 cells were plated with 1.5 × 10^6^ cells/10-cm dish. Cells were infected with Ad-wt *p53* or Ad-*p53* 14/19 and treated with doxorubicin as described above. Cells were lysed in RIPA buffer (50 mM Tris-HCl, 1% NP40, 0.25% sodium deoxycholate, 150 mM NaCl, 1 mM EGTA, 1 mM sodium orthovanadate, 1 mM sodium fluoride). 100 *μ*g of protein from cell lysates was separated by SDS–PAGE. Western blots were stained with antibodies against p53, Mdm2 (both kindly provided by Arnold Levine), phosphorylated p53 (Ser-6, -9, -15, -20, -37 and -46, Cell Signaling Technology, Beverly, MA, USA), phosphorylated Mdm2 (Ser-166, Cell Signaling Technology, Beverly, MA, USA) and Bax (Transduction Laboratories, Lexington, KY, USA). In order to observe the expression of multidrug-resistance proteins in NHF, HT1080 and SJSA, the MDR, MRP1 and MRP4 antibodies (Santa Cruz Biotechnology, Inc., CA, USA) were used. Protein levels were standardized with a monoclonal antibody against glyceraldehyde-3-phosphate dehydrogenase (anti-GAPDH; Chemicon International, Inc., Temecula, CA, USA). Blots were scanned with Image Quant software to detect proteins using an electrochemifluorescence detection system (Amersham Corp., Arlington Heights, IL, USA) on a Molecular Dynamics Storm PhosphorImager (Sunnyvale, CA, USA).

## RESULTS

### Induction of apoptosis and p53 downstream targets by adenovirus-p53 vectors

**Figure 1:**
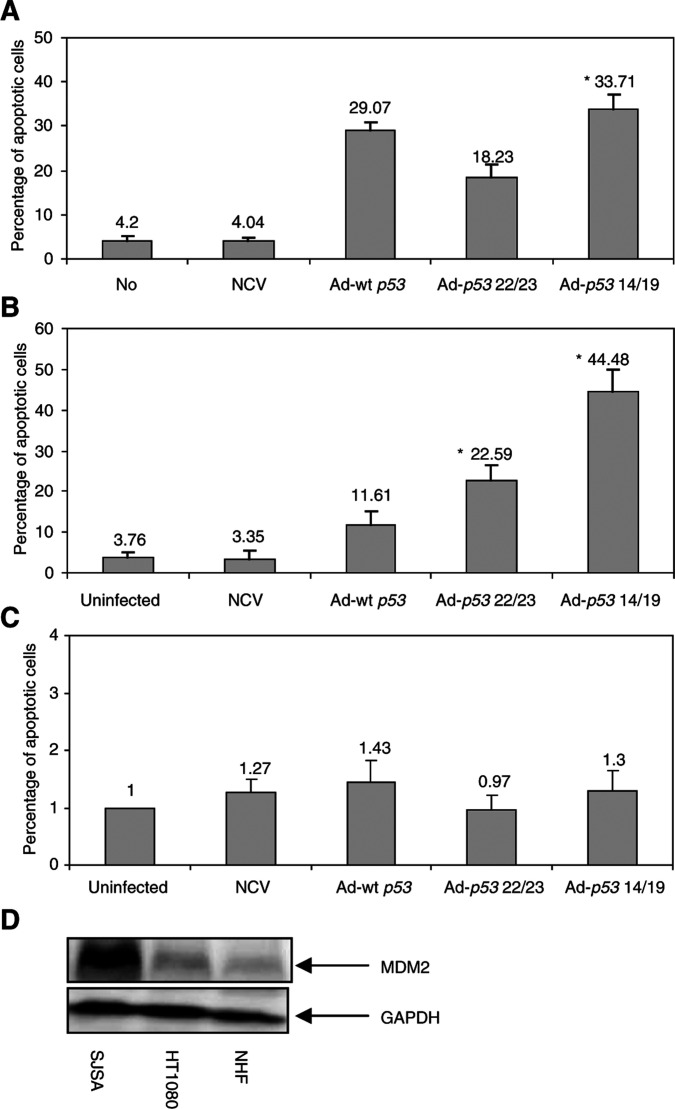
Ad-*p53* 14/19 induces significant apoptosis in sarcoma cell lines. (**A**) HT1080 fibrosarcoma cells, (**B**) SJSA osteosarcoma cells and (**C**) NHF were infected with NCV or Ad-wt *p53*, Ad-*p53* 14/19 or Ad-*p53* 22/23. Cells were stained with propidium iodide (PI), apoptosis was assessed with sub-G1 profile analysis using a FACScan flow cytometer and fold increase of apoptotic cells was calculated. Values shown are the mean+standard deviation of Log [PI] from three independent experiments.^*^ Indicates a significant increase compare to Ad-wt *p*53 treatment (*P*<0.05 in a two-tail Student's *t*-test). (**D**) The expression of Mdm2 in SJSA, HT1080 and NHF. Proteins were extracted from untreated cells and analysed by Western blot using antibodies against Mdm2. GAPDH is the protein loading control.

To determine whether *p53* 14/19 and *p53* 22/23 could induce apoptosis, two human sarcoma cell lines with either low (HT1080) or high (SJSA) levels of Mdm2 were utilized (Figure 1D

). Normal human skin fibroblasts with low levels of Mdm2 were used to assess the toxicity of Ad-*p53* 14/19 and Ad-*p53* 22/23. As shown in Figure 1, apoptosis was significantly induced by Ad-wt *p53* and Ad-*p53* 14/19 and to a lesser degree by Ad-*p53* 22/23 in low Mdm2-expressing HT1080 cells. Apoptotic cells increased seven- to eight-fold after infection with Ad-wt *p53* or Ad-*p53* 14/19 and four-fold after infection with Ad-*p53* 22/23, when compared to uninfected cells or cells infected with NCV (Figure 1A). In contrast, in high Mdm2 expressing SJSA cells, there was a marked difference between Ad-wt *p53* and Ad-*p53* 14/19. There was a four-fold increase in apoptotic cells in SJSA infected with Ad-*p53* 14/19 compared to Ad-wt *p53*, and a two-fold increase of apoptosis in SJSA infected with Ad-*p53* 22/23 compared to Ad-wt *p53* (Figure 1B). No induction of apoptosis was detected in normal fibroblasts after infection by any adenoviral construct (Figure 1C). These results indicate that Ad-*p53* 14/19 and Ad-*p53* 22/23, both defective in Mdm2 binding, can induce apoptosis in HT1080 and SJSA cells regardless of Mdm2 expression, but have minimal effect on normal cells. Ad-*p53* 14/19 induces significant apoptosis in SJSA cells despite the overexpression of Mdm2.

In Figure 2

**Figure 2:**
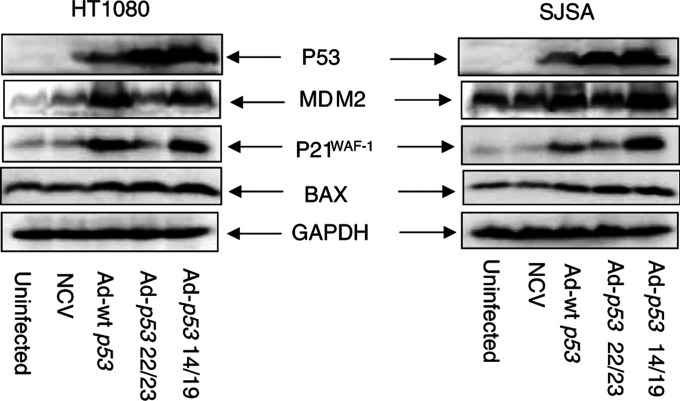
Ad-*p53* 14/19 is a more potent inducer than Ad-*p53* 22/23 for p21^WAF-1^ and Mdm2, but not Bax in HT1080 and SJSA cell lines. Both cell lines were infected with NCV, Ad-wt *p53*, Ad-*p53* 22/23 and Ad-*p53* 14/19. After 24 h, whole-cell proteins were extracted and analysed by Western blot using antibodies against p53, Mdm2, p21^WAF-1^ and Bax, respectively. GAPDH is the protein loading control.

, it is apparent that *p53* 14/19 and *p53* 22/23 have different transcriptional activation activity. The 22/23 mutant has lost its transcriptional activation activity, but the 14/19 mutant retains the activity of the wild-type protein (Lin *et al*, 1994). Ad-*p53* 14/19 induced the expression of p53 target proteins Mdm2, p21^WAF-1^and Bax in both HT1080 and SJSA, and in SJSA there was greater induction of Mdm2 and p21^WAF-1^ than with Ad-wt *p*53. Ad-*p53* 22/23 had the same ability as Ad-*p53* 14/19 to induce Bax, but failed to induce Mdm2 and p21^WAF-1^ in either cell line. Since *p53* 14/19 retains its transactivation capability and induces greater apoptosis than *p53* 22/23 in both HT1080 and SJSA cells, we selected p53 14/19 for further investigation to determine if it could sensitize cells to apoptosis caused by doxorubicin or cisplatin.

### Adenovirus-*p53* 14/19 sensitizes sarcoma cells to doxorubicin and cisplatin

**Figure 3:**
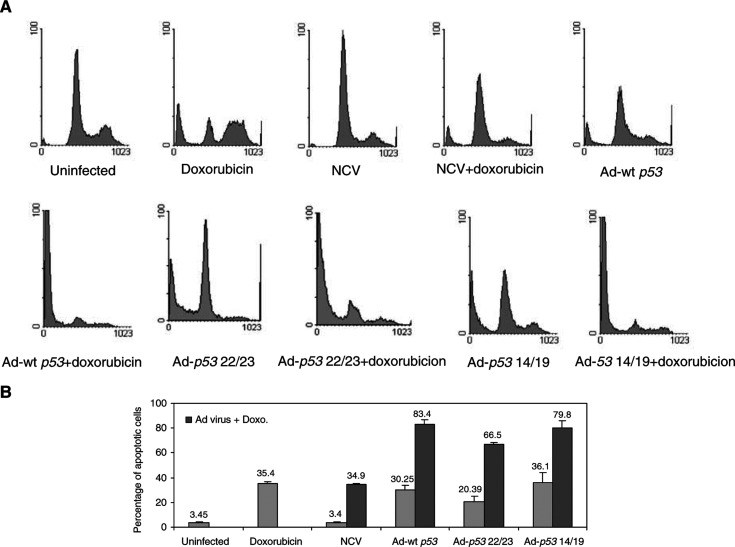
Ad-*p53* 14/19, Ad-*p53* 22/23 and Ad-wt *p53* enhance doxorubicin (Doxo)-mediated apoptosis in HT1080 fibrosarcoma cells. Cells were infected with NCV, Ad-wt *p53*, Ad-*p53* 22/23 or Ad-*p53* 14/19 for 3 days alone or in combination with 0.1 *μ*g/ml doxorubicin. Cells were then stained with PI and apoptosis was assessed with sub-G1 profile analysis using a FACScan flow cytometer. Examples of apoptosis are shown in (**A**). (**B**) Percentages of apoptotis are given as the mean+standard deviation of Log [PI] from three independent experiments. Gray bars indicate HT1080 cells treated by mock infection, doxorubicin, NCV, Ad-wt *p53*, Ad-*p53* 22/23 or Ad-*p53* 14/19 alone. Black bars indicate cells treated with doxorubicin plus NCV, Ad-wt *p53*, Ad-*p53* 22/23 or Ad-*p53* 14/19.

We analysed whether Ad-*p53* 14/19 could sensitize sarcoma cells to apoptosis induced by doxorubicin regardless of Mdm2 expression level. As shown in Figures 3

and 4

**Figure 4:**
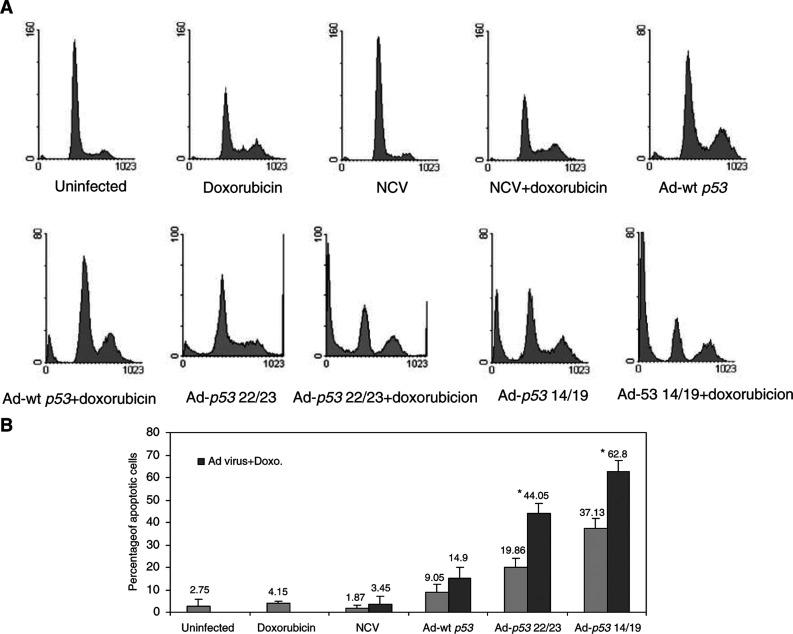
Ad-*p53* 14/19 enhances doxorubicin-mediated apoptosis in SJSA osteosarcoma cells. Cells were infected with NCV, Ad-wt *p53*, Ad-*p53* 22/23 or Ad-*p53* 14/19 for 3 days alone or in combination with 0.1 *μ*g ml^−1^ doxorubicin. Cells were then stained with PI and apoptosis was assessed with sub-G1 profile analysis using a FACScan flow cytometer. Examples of apoptosis in SJSA cells are shown in (**A**). (**B**) Percentages of apoptotic cells are given as the mean+standard deviation of Log [PI] from three independent experiments. ^*^ Indicates a significant increase compared to Ad-wt *p*53 plus doxorubicin treatment (*P*<0.001 in a two-tail Student's *t*-test). Gray bars indicate SJSA cells treated by mock infection, doxorubicin, NCV, Ad-wt *p53*, Ad-*p53* 22/23 or Ad-*p53* 14/19 alone. Black bars indicate cells treated with doxorubicin plus NCV, Ad-wt *p53*, Ad-*p53* 22/23 or Ad-*p53* 14/19.

, HT1080 is relatively sensitive and SJSA relatively resistant to doxorubicin treatment. After exposure to 0.1 μg ml^−1^ of doxorubicin, 35% of HT1080 cells were apoptotic (Figure 3B), while only 4% of SJSA cells were apoptotic (Figure 4B). When doxorubicin was combined with Ad-wt *p53*, Ad-*p53* 22/23 or Ad-*p53* 14/19, 66–83% of HT1080 cells underwent apoptosis (Figure 3B). However, in SJSA cells, when doxorubicin was combined with Ad-wt *p53*, only 15% of cells were apoptotic (Figure 4B), demonstrating continued resistance. In contrast, doxorubicin plus Ad-*p53* 22/23 or Ad-*p53* 14/19 generated greater apoptosis (44 or 63%) in SJSA cells. This demonstrates that Ad-*p53* 22/23 and Ad-*p53* 14/19 can not only enhance apoptosis induced by doxorubicin in sensitive cells such as HT1080 but can also overcome Mdm2-mediated doxorubicin resistance, as in SJSA cells. Moreover, the results suggest that Ad-*p53* 14/19 has more significant synergy with doxorubicin than Ad-*p53* 22/23.

**Figure 5:**
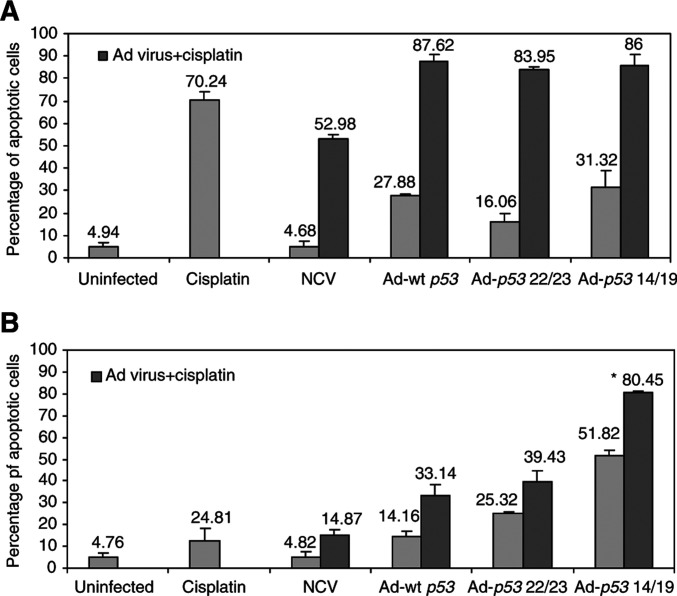
Ad-*p53* 14/19 combined with cisplatin induces more significant apoptosis than Ad-wt *p53* combined with cisplatin in SJSA cells. Cells were infected with NCV, Ad-wt *p53*, Ad-*p53* 22/23 or Ad-*p53* 14/19 for 3 days alone or in combination with 5 μg/ml cisplatin. Cells were then stained with PI and apoptosis was assessed with sub-G1 profile analysis using a FACScan flow cytometer. Percentages of apoptosis in HT1080 cells are shown in (**A**), and percentages of apoptosis in SJSA cells are shown in (**B**). Values shown are the mean+standard deviation of Log [PI] from three independent experiments. ^*^ Indicates a significant increase compared to Ad-wt *p*53 plus cisplatin treatment (*P*<0.005 in a two-tail Student's *t*-test). Gray bars indicate SJSA cells treated by mock infection, cisplatin, NCV, Ad-wt *p53*, Ad-*p53* 22/23 or Ad-*p53* 14/19 alone. Black bars indicate cells treated with cisplatin plus NCV, Ad-wt *p53*, Ad-*p53* 22/23 or Ad-*p53* 14/19.

In order to further demonstrate that Ad-*p53* 14/19 can increase apoptosis induced by a chemotherapeutic agent, cisplatin was selected. Cisplatin is a common chemotherapeutic agent for sarcomas, such as osteosarcoma (Stine *et al*, 2003). As shown in Figure 5

, HT1080 is still relatively more sensitive to cisplatin treatment than SJSA. After exposure to 5 *μ*g ml^−1^ of cisplatin, 70% of HT1080 cells were apoptotic (Figure 5A), while only 24% of SJSA cells were apoptotic (Figure 5B). When cisplatin was combined with Ad-wt *p53*, Ad-*p53* 22/23 or Ad-*p53* 14/19, around 84–88% of HT1080 cells underwent apoptosis (Figure 5A). However, in SJSA cells, when cisplatin was combined with Ad-wt *p53* and Ad-*p53* 22/23, only 33–39% of cells were apoptotic (Figure 5B). In contrast, cisplatin plus Ad-*p53* 14/19 generated greater apoptosis (80%) in SJSA cells. This demonstrates that Ad-*p53* 14/19 can not only enhance apoptosis induced by doxorubicin but can also enhance apoptosis induced by cisplatin in either HT1080 or SJSA cells.

### Doxorubicin induces phosphorylation of wt p53 and p53 14/19 proteins

**Figure 6:**
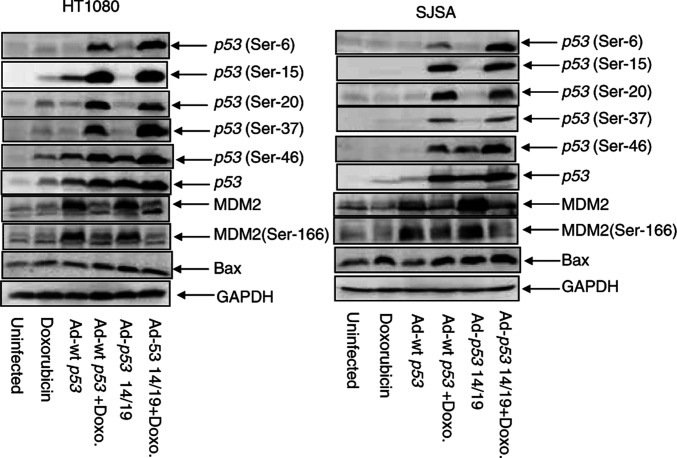
Doxorubicin induces phosphorylation of p53 in HT1080 (low Mdm2) and SJSA (high Mdm2). Cells were treated with Ad-*p53* or Ad-*p53* 14/19 and 0.1 *μ*g/ml doxorubicin (Doxo). After 24 h, whole-cell proteins were extracted and analysed by Western blot using antibodies against p53, phosphorylated p53 (Ser-6, Ser-15 Ser-20, Ser-37 and Ser-46), Mdm2, phosphorylated Mdm2 (Ser-166) and Bax. GAPDH is the protein loading control.

To evaluate phosphorylation of p53 and expression of its target proteins, SJSA and HT1080 were infected with Ad-wt *p53* or Ad-*p53* 14/19 in conjunction with exposure to 0.1 *μ*g ml^−1^ doxorubicin. Western blotting showed that expression of p53 was induced in cells treated with both Ad-wt *p53* plus and Ad-*p53* 14/19 plus doxorubicin (Figure 6

). In SJSA cells, Ad-*p53* 14/19 alone resulted in a higher p53 expression and greater induction of downstream targets Mdm2 and Bax than Ad-wt *p53*. In HT1080 cells, both Ad-*p53* 14/19 and Ad-wt *p53* induced p53 target proteins Mdm2 and Bax. Mdm2 was phosphorylated in cells infected with Ad-*p53* 14/19 and Ad-wt *p53* alone.

In both HT1080 and SJSA cells, there was slightly higher p53 expression in cells treated with Ad-*p53* 14/19 plus doxorubicin than in cells treated with Ad-wt *p53* plus doxorubicin (Figure 6). This suggests that doxorubicin stabilizes wt p53 protein and may further stabilize p53 14/19. In both cell lines treated with Ad-wt *p53* plus doxorubicin or Ad-*p53* 14/19 plus doxorubicin, there were dramatic increases in p53 phosphorylation at Ser-6, Ser-15, Ser-20, Ser-37 and Ser-46. p53 phosphorylation mediated by doxorubicin may enhance Bax (Figure 6) and p21^WAF-1^ (data not shown) induction in SJSA cells. This serine phosphorylation is weakly or not detected in cells infected with Ad-wt *p53* or Ad-*p53* 14/19 alone or cells treated with doxorubicin alone. In SJSA cells, Mdm2 was slightly increased in Ad-wt *p53*-infected cells, but was dramatically increased in Ad-*p53* 14/19-infected cells. In both cell lines, Mdm2 expression and phosphorylation were decreased in cells treated with doxorubicin alone or Ad-wt *p53* plus doxorubicin, and markedly diminished in cells infected with Ad-*p53* 14/19 plus doxorubicin (Figure 6).

## DISCUSSION

Derangements of the *p53* pathway are common in soft-tissue sarcomas and osteosarcomas. Mdm2 overexpression occurs frequently, and these tumours typically contain wt *p53* (Oliner *et al*, 1992). Since Mdm2 inactivates and promotes degradation of p53, the ability to restore p53 function by introduction of wt *p53* into sarcomas is limited. We tested two modified *p53* genes that are not responsive to the Mdm2 autoregulatory feedback loop (Lin *et al*, 1994). Ad-*p53* 22/23, an adenovirus vector encoding a transcription-defective p53 mutant, is able to induce limited apoptosis compared to Ad-wt *p53* in both sarcoma cell lines tested, even combined with doxorubicin or cisplatin. Since both cell lines express endogenous wt *p53* (Evdokiou and Cowled, 1998), it is possible that p53 22/23 may form dimers or tetramers with endogenous wt p53. These hetero-tetramers may retain partial function as a transcription factor. Interestingly, Ad-*p53* 22/23, although unable to induce Mdm2 and p21^WAF-1^, can still induce Bax expression in these cell lines. Bax is one of the p53 primary-response genes involved in the induction of apoptosis (Miyashita and Reed, 1995); this may explain in part why Ad-*p53* 22/23 is able to induce limited apoptosis in these cell lines. In addition to induction of apoptosis by transcription-dependent mechanisms, transcription-independent mechanism(s) have been reported for p53 (Haupt *et al*, 1997b; Yan *et al*, 1997; Mihara *et al*, 2003). We observed that Ad-*p53* 22/23 failed to induce apoptosis in the Caov-3 cell line, but induced limited apoptosis in MDAH 2774 cell line (data not shown). Both ovarian cancer cell lines harbour p53 mutations (Yaginuma and Westphal, 1992). Therefore, *p53* 22/23 may still be able to induce apoptosis via a transcription-independent mechanism(s).

*p53* 14/19 maintains its transcriptional activation and antiproliferative functions in the face of high Mdm2 levels (Chen *et al*, 1995, 1996). Our data indicate that *p53* 14/19 has greater ability to activate Mdm2 and p21^WAF-1^ than *p53* 22/23, and induces more significant apoptosis than *p53* 22/23 in sarcoma cells. These results suggest that *p53* 14/19 would be the better construct to use in further research targeting sarcoma cell lines with high levels of Mdm2. In addition, *p53* 14/19 more dramatically induced p21^WAF-1^ than wt *p*53 in SJSA cells, but there was equivalent induction of p21^WAF-1^ in HT1080 cells. There appears to be no relationship between the induced level of p21^WAF-1^ and the induced apoptosis by wt *p*53 or *p53* 14/19 in HT1080 or SJSA, suggesting that the induction of p21^WAF-1^ is not responsible for p53-dependent apoptosis in this model system. The major function of p21^WAF-1^ is to induce cell cycle growth arrest at the G1 phase (Medrano *et al*, 1995). Therefore, it is likely that other downstream targets of p53 are more directly involved with induction of apoptosis induced by p53 14/19.

We have shown that Ad-*p53* 14/19 induces dramatic apoptosis in sarcoma cell lines with either low or high Mdm2 expression. In HT1080 cells (low Mdm2), Ad-*p53* 14/19 induced apoptosis similar to Ad-wt *p53*. In SJSA cells (high Mdm2), Ad-*p53* 14/19 induced dramatically more apoptosis than Ad-wt *p53* (44 *vs* 11%). These results are consistent with previous Ad-*p53* 14/19 data from cell growth inhibition assays (Lin *et al*, 2000). We also observed that Ad-*p53* 14/19 more potently inhibits SJSA cells’ growth than Ad-wt *p53* in nude mice (Tang and Lin, unpublished data). Neither construct appeared to be toxic to normal skin fibroblasts *in vitro*. Two possible explanations for this selectivity are that cancer cells may be more sensitive to adenoviral transfer of wt *p53*, *p53* 22/23 or *p53* 14/19, or that oncogenes such as c-*myc* and cyclin D1 are overexpressed in malignant but not normal cells. Overexpression of these oncogenes sensitizes cells to apoptosis induced by wt *p53* (Hermeking and Eick, 1994; Wagner *et al*, 1994; Lin *et al*, 1996; Hiyama and Reeves, 1999). We observed no evidence that adenoviral transfer of *p53* 14/19 would be any less safe than transfer of wt *p53* to normal cells.

Chemoresistance is a major problem in the treatment of malignant tumours; hence, it will be of potential value to discover combination protocols that overcome resistance. Doxorubicin, a topoisomerase II inhibitor, is commonly used in the treatment of sarcomas (Sondak and Chang, 2001). Doxorubicin causes apoptosis by direct DNA damage, at least in part in a p53-dependent manner (Lowe *et al*, 1993, 1994; Malcomson *et al*, 1995; Vikhanskaya *et al*, 1997; Weinstein *et al*, 1997; Blagosklonny and El-Deiry, 1998). Cisplatin is also one of the most widely used chemotherapeutic agents for the treatment of many types of cancer. Apoptosis is the primary mode of cell death induced by cisplatin, and cisplatin occurs primarily through its ability to bind covalently to DNA and prevent DNA replication and transcription (Li *et al*, 1998; Masuda *et al*, 1998). The HT1080 cell line is sensitive to doxorubicin (Li *et al*, 2001), and the present study shows that HT1080 is also more sensitive to cisplatin. We found that SJSA was resistant to doxorubicin and not more sensitive to cisplatin than HT1080. These results are in agreement with one previous report that the *mdm2* gene caused resistance to doxorubicin but not to cisplatin in some sarcoma cell lines, and in these lines there was an increase in the expression of the *mdr-1* gene that encodes P-gp (Cocker *et al*, 2001). However, we did not observe the expression of multidrug resistance proteins, such as MDR, MRP1 and MRP4 in NHF, HT1080 and SJSA cell lines (data not shown). Our data suggest that Ad-*p53* 14/19, but not Ad-wt *p53* is able to overcome this resistance to doxorubicin as well as enhance SJSA cells’ sensitivity to cisplatin. Nevertheless, Ad-*p53* 22/23 is also able to increase apoptosis induced by doxorubicin in SJSA cells’ sensitize has the same effect as Ad-wt *p53* in enhancing apoptosis induced by cisplatin (Figure 4B and 5B). Ad-*p53* 14/19 and Ad-*p53* 22/23 also augmented the apoptotic response to doxorubicin and cisplatin in HT1080, the sensitive cell line (Figure 3B and 5A).

p53 phosphorylation is induced by DNA damage at a variety of sites including Ser-6, Ser-15, Ser-20, Ser-37 and Ser-46 (Sakaguchi *et al*, 1998; Tibbetts *et al*, 1999; Higashimoto *et al*, 2000; Oda *et al*, 2000; Achanta *et al*, 2001; Shono *et al*, 2002). These modifications may stabilize and activate p53 as a transcription factor. (Shieh *et al*, 1997; Siliciano *et al*, 1997; Chehab *et al*, 2000). Our studies show that both Ad-wt *p53* and Ad-*p53* 14/19 in combination with doxorubicin dramatically increase p53 phosphorylation. Previous studies have shown that phosphorylation at Ser-15, Ser-20 and Ser-46 is critical for regulating apoptotic activity (Oda *et al*, 2000; Shono *et al*, 2002). Phosphorylation of Ser-15, Ser-20 and Ser-37 interferes with Mdm2 interaction and impairs the ability of Mdm2 to inhibit p53-dependent transactivation (Shieh *et al*, 1997; Chehab *et al*, 1999; Appella and Anderson, 2000). We found that both wt *p53* and *p53* 14/19 are phosphorylated at Ser-6, Ser-15 Ser-20, Ser-37 and Ser-46 in response to doxorubicin. Hence, the mutations at residues 14 and 19 do not affect these phosphorylation sites. Interestingly, levels of Ser-6 and Ser-46 phosphorylation of *p53* 14/19 were much higher than wt *p53* in SJSA cells.

Infection with Ad-wt *p53* combined with doxorubicin resulted in enhanced apoptosis compared to Ad-wt *p53* or doxorubicin alone in HT1080 but not in SJSA cells. This suggests that negative regulation of p53 by Mdm2 in SJSA cells may limit the magnitude of p53 activation and p53-dependent apoptosis even when p53 is phosphorylated. Mdm2 overexpression diminished the apoptotic response to doxorubicin even when exogenous wild-type p53 was reintroduced, consistent with other reports (Cocker *et al*, 2001).

Doxorubicin treatment reduced Mdm2 expression and phosphorylation in both cell lines. This is consistent with a previous report that Mdm2 is downregulated by anticancer drugs including doxorubicin (Gao *et al*, 1999). Doxorubicin can activate p53 in Mdm2-overexpressing cells by decreasing Mdm2 levels, alleviating the inhibition of p53.

We observed increased expression of Bax in SJSA cells treated with Ad-wt *p53* plus doxorubicin compared to cells infected with Ad-wt *p53* alone. Bax levels correlated with p53-induced apoptosis. In HT1080 cells, although the expression of Bax was induced by Ad-wt *p53* or Ad-*p53* 14/19, there was no relationship between Bax expression and apoptosis. Previous studies have shown that Bax induction by p53 is necessary to inhibit tumour growth (Yin *et al*, 1997), but that the contribution of Bax to p53-mediated apoptosis is cell-type dependent (Knudson *et al*, 1995; McCurrach *et al*, 1997). In HT1080 cells, other downstream targets of p53 may be more important for induction of apoptosis.

In conclusion, Mdm2 overexpression can promote cancer cell resistance to DNA-damaging agents and limit the effectiveness of chemotherapeutic drugs. Modified *p53*, particularly *p53* 14/19, retains the proapoptotic and transcriptional activity of wt *p53*, and can augment the effectiveness of chemotherapy even in cells overexpressing Mdm2. Strategies involving *p53* 14/19 with chemotherapeutic agents may be a useful approach for sarcomas and other tumours with high levels of Mdm2.
